# A review of AI-based radiogenomics in neurodegenerative disease

**DOI:** 10.3389/fdata.2025.1515341

**Published:** 2025-02-20

**Authors:** Huanjing Liu, Xiao Zhang, Qian Liu

**Affiliations:** ^1^The Department of Applied Computer Science, Faculty of Science, University of Winnipeg, Winnipeg, MB, Canada; ^2^The Department of Biochemistry and Medical Genetics, Max Rady College of Medicine, Rady Faculty of Health Sciences, University of Manitoba, Winnipeg, MB, Canada

**Keywords:** radiogenomics, neurodegenerative disease, artificial intelligence, medical image, multi-omics, deep learning

## Abstract

Neurodegenerative diseases are chronic, progressive conditions that cause irreversible damage to the nervous system, particularly in aging populations. Early diagnosis is a critical challenge, as these diseases often develop slowly and without clear symptoms until significant damage has occurred. Recent advances in radiomics and genomics have provided valuable insights into the mechanisms of these diseases by identifying specific imaging features and genomic patterns. Radiogenomics enhances diagnostic capabilities by linking genomics with imaging phenotypes, offering a more comprehensive understanding of disease progression. The growing field of artificial intelligence (AI), including machine learning and deep learning, opens new opportunities for improving the accuracy and timeliness of these diagnoses. This review examines the application of AI-based radiogenomics in neurodegenerative diseases, summarizing key model designs, performance metrics, publicly available data resources, significant findings, and future research directions. It provides a starting point and guidance for those seeking to explore this emerging area of study.

## 1 Introduction

Neurodegeneration refers to the progressive loss of neuron structure or function, impairing neurological processes such as movement, memory, and cognition (Ueha et al., 2024; Feng, 2023). Globally, neurodegenerative diseases significantly increase mortality and morbidity, especially among the elderly (Erkkinen et al., 2018). There are roughly five categories of neurodegenerative diseases, including Multiple Sclerosis (MS), Dementia, Parkinson's Disease (PD), Amyotrophic Lateral Sclerosis (ALS), and Creutzfeldt-Jakob Disease. MS is a chronic demyelinating disease affecting the central nervous system (Bae et al., 2023), which represents demyelinating diseases in the neurodegenerative category (Przedborski et al., 2003). It involves an abnormal immune response against the myelin sheath, disrupting nerve signal transmission and causing symptoms like fatigue, motor dysfunction, and cognitive impairment. MS is typically diagnosed in early adulthood, with 65% of new diagnoses occurring in Canadians aged 20–49 years (Public Health Agency of Canada, 2018a). MS is characterized by hyperintense lesions in specific brain regions and is linked to HLA-DRB1, IL7R, and IL2RA genes. Dementia, including Alzheimer's Disease (AD), Dementia with Lewy Bodies (DLB), and Frontotemporal Dementia (FTD), significantly affects memory and cognitive abilities (Arvanitakis et al., 2019). Over 402,000 seniors in Canada are living with dementia, with an incidence rate of 14.3 new cases per 1,000 seniors annually (Public Health Agency of Canada, 2017). AD is characterized by amyloid-beta plaques and neurofibrillary tau tangles in the brain, leading to neuronal loss and cognitive decline (Jahn, 2013; Migliore and Coppedè, 2022; Rajmohan and Reddy, 2017). AD shows hippocampal atrophy, widened sulci, enlarged ventricles, and is associated with gene APOE, APP, PSEN1, PSEN2, and TREM2 (Andrade-Guerrero et al., 2023; Wolfe et al., 2018). PD is characterized by motor symptoms such as tremors and rigidity (Little et al., 2012). It results from the loss of dopamine-producing neurons in the substantia nigra (Damier et al., 1999). PD affects ~84,000 Canadians aged 40 and older, with an incidence rate of 55.1 per 100,000 population (Public Health Agency of Canada, 2018b). Non-motor symptoms in advanced stages include cognitive dysfunction, psychiatric changes, and sensory symptoms (Szymański et al., 2010; Jankovic et al., 2021). PD involves atrophy in the substantia nigra and iron deposition in the basal ganglia, with genetic markers like SNCA, LRRK2, PARK2, PINK1, DJ-1, and α-synuclein protein. ALS, or Lou Gehrig's disease, is a progressive disorder affecting motor neurons, leading to muscle weakness, atrophy, and paralysis (Morris, 2015). While most ALS cases are sporadic, about 10% are familial (Zou et al., 2017). ALS progresses rapidly, with a median survival time of 3 to 5 years post-diagnosis (Testa et al., 2004). Recent studies report an ALS incidence rate of 2.13 per 100,000 in Nova Scotia and 2.4 per 100,000 in Newfoundland and Labrador (Wolfson et al., 2009). ALS features atrophy in the motor cortex and hyperintense signals in corticospinal tracts, which are linked to SOD1, C9orf72, TARDBP, and FUS, and involve FUS, TDP-43, and SOD-1 proteins. CJD is a rare, fatal disorder caused by prions, leading to rapid brain damage, cognitive decline, and motor dysfunction (Head and Ironside, 2012). CJD progresses swiftly, often resulting in death within a year of symptom onset (Josephs et al., 2009). In 2022, 90 people in Canada were diagnosed with CJD, with an incidence rate of 1 to 2 per million annually (Public Health Agency of Canada, 2023). Lastly, CJD is marked by hyperintense signals in the basal ganglia, thalamus, and cortical regions, associated with the PRNP gene and the PrPSc protein.

**Figure 1:**
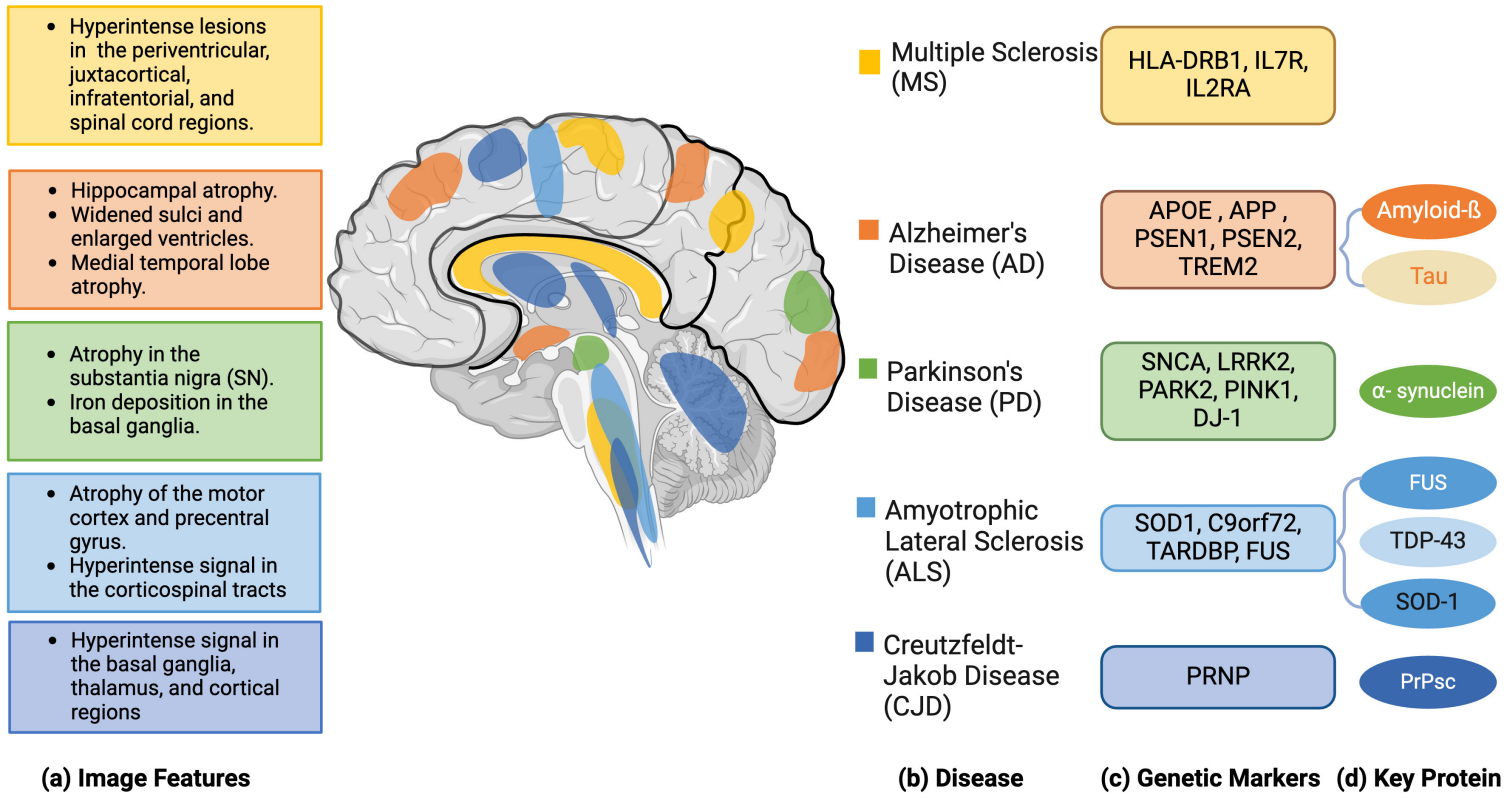
Neuroimaging features, genetic markers, and key proteins associated with five representative neurodegenerative diseases.

With aging as the primary risk factor, the prevalence of these diseases continues to rise. Although several medications manage symptoms, effective treatments remain limited, making early diagnosis crucial (Hou et al., 2019; Lamptey et al., 2022). Traditional diagnosis of neurodegenerative diseases relies on image features obtained from medical imaging techniques such as X-ray, Magnetic Resonance Imaging (MRI), Computed Tomography (CT), Positron Emission Tomography (PET), and Single-Photon Emission Computed Tomography (SPECT). These methods are effective at capturing morphological and phenotypical changes in the brain. However, imaging features frequently overlap among different neurodegenerative conditions, for example, gray matter atrophy patterns in AD and FTD can appear similar in certain brain regions, making accurate diagnosis complicated (Frings et al., 2014; Whitwell et al., 2017; Mazón et al., 2018). Moreover, medical images struggle with early detection, often revealing issues only after disease progression (Bevilacqua et al., 2023). Integrating medical images with genomic and clinical data could provide a more comprehensive and earlier diagnosis (Bevilacqua et al., 2023). Genomics is the study of the complete set of genetic material, focusing on the structure, function, and evolution of genomes (Zhang, 2023). This includes analyzing DNA sequences, genetic variants, DNA methylation patterns, mRNA expression to understand the complex interactions that govern biological processes. It may reveal the potential risk of neurodegenerative disease even before the early onset, when there are no obvious changes in imaging data. In addition, genomics enhances our understanding of the molecular mechanisms driving phenotypical changes in neurodegenerative diseases, and it may help identify key biomarkers that could enable personalized treatment approaches tailored to an individual's genetic makeup (Liu et al., 2023). Figure 1 shows five representative neurodegenerative diseases with their image and genomics features.

Radiogenomics is an emerging field that studies the relationship between imaging features and genetic variations. It aims to link specific genomic markers with characteristics observed in medical images, helping to understand how genetic factors influence disease development and progression (Shui et al., 2021). This approach can offer deeper insights into neurodegenerative disease mechanisms and may help improve diagnosis, prognosis, and treatment strategies (Mazurowski, 2015). A key challenge in radiogenomics is the integration of multi-modal, high-dimensional imaging and genomic data. The complexity often introduces significant noise and confounding factors, making it difficult to extract meaningful biological insights and accurately interpret the relationships between genetic variations and imaging phenotypes. To address these issues, artificial intelligence (AI) methods including Machine Learning (ML), Deep Learning (DL), and Natural Language Processing (NLP), are increasingly applied to analyze medical images and genomics data for neurodegenerative diseases (Feng, 2023). Recent advancements in computer science, particularly DL, have revolutionized neurodegenerative disease research. DL models, such as convolutional neural networks (CNNs), fully connected neural networks (FNNs), generative adversarial networks (GANs), auto-encoders, deep belief networks (DBNs), and recurrent neural networks (RNNs), have demonstrated exceptional capabilities in analyzing complex neuroimaging and genomics data.

This review outlines the application of DL-based radiogenomics in neurodegenerative diseases, highlighting key model architectures, performance evaluation metrics, publicly accessible data sources, significant discoveries, and potential future research directions. It serves as a valuable resource and guide for researchers interested in exploring this evolving field.

## 2 Database

Several publicly available data resources support radiomics, genomics, and radiogenomics studies for neurodegenerative diseases. Table 1 provides key information about these resources, including their data types, along with links to access them.

**Table 1:** Overview of biomedical datasets with imaging and/or genomics data.

**Dataset**	**Disease**	**Image**	**Omics**	**Link**
Alzheimer's Disease Neuroimaging Initiative (ADNI)	AD, LBD, FTD, Parkinson's disease dementia (PDD), etc.	MRI, PET, Cerebrospinal Fluid (CSF) biomarker imaging, Resting-State functional MRI (rs-fMRI), etc.	Genome-Wide Association Study (GWAS) data, Whole-Genome Sequencing (WGS) data, Whole-Exome Sequencing (WES) data, RNA sequencing (RNA-seq) data, DNA methylation data, MicroRNA (miRNA) expression data, APOE genotyping data, Copy Number Variation (CNV) data, Polygenic Risk Score (PRS) data, Somatic mutation data, Proteomics data, Metabolic profile data	https://adni.loni.usc.edu
UK biobank	AD, PD, ALS, etc.	MRI, fMRI, X-ray, Ultrasound, etc.	Genotyping data (SNPs), WES data, GWAS data, PRS data, CNVs, DNA methylation	https://www.ukbiobank.ac.uk/
National Alzheimer's Coordinating Center (NACC)	AD, LBD, PD, HD, ALS, FTD, etc.	MRI, PET	Genotyping data (SNPs), WES data, WGS data, PRS data, CNVs	https://naccdata.org/
Enhancing Neuroimaging Genetics through Meta-Analysis (ENIGMA)	AD, PD, MS, FTD, DLB, etc.	MRI, fMRI	GWAS data, PRS data, DNA methylation, Histone modification data, CNVs, Transcriptomic data, Rare variant analysis	https://enigma.ini.usc.edu/
Wisconsin Registry for Alzheimer's Prevention (WRAP)	AD	MRI, PET, fMRI	GWAS data, PRS data, DNA methylation, RNA sequencing	https://wrap.wisc.edu/
Australian Imaging, Biomarker and Lifestyle Flagship Study of Aging (AIBL)	AD	MRI, PET	GWAS data, PRS data, DNA methylation	https://aibl.org.au/
Parkinson's Progression Markers Initiative (PPMI)	PD	MRI, DAT-SPECT	GWAS data, PRS data, DNA methylation	https://www.ppmi-info.org/
Diagnosis and Monitoring of Prion Diseases (DOMP)	Prion disease	MRI, fMRI	WGS data, Targeted gene sequencing, DNA methylation	N/A
Gene Expression Omnibus (GEO)	AD, PD, DLB, etc.		Gene expression data, Epigenomic data, Genomic Variants data, Proteomics data, Metabolomics data	http://www.ncbi.nlm.nih.gov/geo/
National Center for Geriatrics Gerontology (NCGG)	AD, PD, LBD, etc.	MRI, PET, fMRI	GWAS data, WGS data, DNA methylation, RNA sequencing, PRS data	https://www.ncgg.go.jp/research/
Open Access Series of Imaging Studies (OASIS)	AD	MRI, fMRI, PET		http://www.oasis-brains.org/
Kaggle dataset	AD, PD, etc.	MRI, PET	Gene expression data, SNPs	http://www.kaggle.com/tourist55/alzheimers-dataset-4-class-of-images

ADNI (Jack et al., 2008) is a global, multisite longitudinal study involving over 1,500 participants across 63 sites in the US and Canada, tracking AD progression from normal aging through stages of cognitive impairment to dementia. The dataset includes clinical assessments, brain imaging (MRI, PET), genetic data, and biospecimen biomarkers. ADNI's goal is to enhance early diagnosis and treatment by identifying and validating biomarkers, making it a crucial resource that has significantly improved diagnostic methods, clarified disease mechanisms, and supported new treatment development. UK Biobank (Sudlow et al., 2015) is a comprehensive biomedical resource containing de-identified genetic, lifestyle, and health data from 500,000 UK participants. Since 2006, it has expanded to include imaging data such as brain, heart, and full-body MRIs, with a goal of imaging 100,000 participants. Genetic data encompasses whole genome and exome sequencing, while health records link to a range of electronic health data. The resource also includes biomarker data, physical activity monitoring, online questionnaires, and biological samples like blood, urine, and saliva. Among these participants, tens of thousands have been diagnosed with neurodegenerative diseases, including Alzheimer's, Parkinson's, and vascular dementia. Data types specifically related to neurodegenerative disease research include brain imaging (structural and functional MRI), cognitive assessments, genotyping for risk alleles, and biomarker data related to neurodegeneration. The NACC (Beekly et al., 2007) dataset is a vital resource for AD research, containing extensive clinical, cognitive, imaging, and neuropathological data collected from AD Research Centers across the U.S. The dataset includes demographic information, medical history, neuropsychological test results, MRI scans, and autopsy findings, providing a rich foundation for developing diagnostic models and studying the progression of AD and related dementias. This comprehensive dataset supports research efforts to improve diagnosis and treatment strategies for neurodegenerative diseases. The ENIGMA (Garg et al., 2015) project is a global collaboration that uses brain imaging and genetic data to study neurological disorders like PD and FTD. It provides T1 MRI scans and conducts meta-analyses of brain structure, focusing on volumetric and shape changes in subcortical regions. For PD, the data include 116 male and 68 female healthy controls (HCs), and 264 male and 142 female PD patients. ENIGMA also analyzes genomics data, such as copy number variations (CNVs), involving ~17,000 individuals.

The WRAP (Johnson et al., 2018) is a longitudinal study primarily focused on identifying risk factors and early biomarkers for AD. It includes over 1,500 participants, most of whom are cognitively healthy but have a family history of AD. The dataset contains neuroimaging data (MRI, PET), genetic information, blood-based biomarkers, cerebrospinal fluid analysis, cognitive performance tests, and lifestyle factors such as physical activity, sleep, and diet. WRAP aims to better understand the early biological and environmental factors contributing to AD development and progression. The AIBL (Ellis et al., 2009) is a comprehensive longitudinal study focused on AD and related dementias. It includes data from over 1,100 participants, consisting of AD patients, individuals with MCI, and HCs. The dataset comprises neuroimaging (MRI, PET), biomarkers (blood, cerebrospinal fluid), genetic data, cognitive assessments, and lifestyle factors such as diet and exercise. AIBL's primary aim is to identify biomarkers and risk factors associated with AD progression, making it a valuable resource for research on neurodegeneration and cognitive decline. The PPMI dataset (Brumm et al., 2023) originates from a longitudinal observational study that evaluated people with PD, including high risk individuals and healthy individuals. The dataset includes clinical, imaging, omics, genetic, sensor, and biomarker data. The sample of participants includes 902 PD and 237 HCs. The DOMP dataset focuses on rare neurodegenerative prion diseases like CJD and others. It includes clinical, neuroimaging (MRI), genetic, and biochemical data, along with neuropathology and longitudinal cognitive assessments. While the sample size is relatively small, typically in the hundreds, the dataset is a valuable resource for tracking disease progression and enhancing diagnostic tools.

In addition, there are some genomics only dataset or images only dataset. The GEO (Barrett et al., 2013) is a public repository that archives and freely distributes high-throughput gene expression and other functional genomics data submitted by the research community. In the GEO database, over 400 datasets focus on neurodegenerative diseases, providing a wealth of data types for in-depth research. These include a range of transcriptomic data from microarrays and RNA sequencing to single-cell transcriptomic profiles that examine specific brain regions and cell types associated with neurodegenerative conditions such as Alzheimer's, Parkinson's, Huntington's disease, and ALS. This variety supports research ranging from identifying disease-specific gene expression profiles to analyzing epigenetic patterns, like DNA methylation and chromatin accessibility, essential for understanding disease mechanisms and progression. The NCGG (National Center for Geriatrics, 2024) is a longitudinal, multisite study primarily focusing on aging populations in Japan, with over 10,000 participants tracked across various time points. This dataset includes comprehensive clinical assessments, advanced imaging data (MRI, PET, DXA), and a rich array of genetic and molecular data, including genomics, proteomics, and metabolomics. NCGG aims to identify key biomarkers related to age-related conditions like Alzheimer's and dementia, osteoporosis, and cardiovascular disease, thus playing a significant role in advancing diagnostic accuracy, understanding disease mechanisms, and guiding therapeutic developments in geriatric health. The OASIS (LaMontagne et al., 2019) is a publicly available dataset aimed at providing neuroimaging data for the scientific community. This initiative is designed to support research in brain imaging, particularly in the study of AD and cognitive aging. OASIS provides high-quality MRI data that includes structural brain images, clinical and cognitive assessments. The data covers a wide range of ages and includes both healthy subjects and individuals with cognitive impairments. The Kaggle dataset (Kaggle, 2024) is a vast collection of datasets available on the Kaggle platform, which is a popular community for data science and machine learning. These datasets cover a wide range of topics including healthcare, finance, social sciences, and image processing, among others. The data types include structured data (e.g., CSV files), unstructured data (e.g., text), time series, images, and more.

## 3 AI in neurodegeneration

### 3.1 Machine learning in neurodegeneration

ML is a subtype of AI that aims to enable models to identify patterns by learning from experience and improving their future predictions over time (Bishop and Nasrabadi, 2006). Unlike traditional heuristic models in clinical medicine, ML methods are based on statistical theory and are designed to be broadly applicable across various types of problems (Duda et al., 2001). ML can be classified as supervised, unsupervised, or reinforcement learning based on the type of outcomes desired from the algorithms. In supervised learning, algorithms learn from labeled data to map inputs to outputs. Unsupervised learning involves finding patterns or structures in unlabeled data, as the algorithm does not predict specific outcomes. Reinforcement learning involves training algorithms in dynamic environments where learning occurs using a system of rewards and penalties, improving performance based on experience rather than predefined data. Table 2 lists the studies that involve machine learning (ML) methods in neurodegenerative diseases.

**Table 2:** Overview of ML methods applied in neurodegenerative disease.

**References**	**Disease**	**Genomics**	**Image**	**Database**	**ML Method**	**Sample**	**Acc**
Shigemizu et al. (2019)	DLB	gene expression, SNPs		NCGG Biobank	Penalized regression, RF, SVM	478	0.829
Singh et al. (2019)	AD, PD		fMRI	ADNI, PPMI	Least-square SVM	2,540	0.8789
Lin et al. (2022)	AD	Gene expression		ADNI	RF	577	0.919
Alatrany et al. (2023)	AD	SNPs		ADNI, AD GWAS, AdaptMap	RF, CNN, TL, SVM	399 from ADNI 364 from AD GWAS	0.89
Hajianfar et al. (2023)	PD		DAT-SPECT	PPMI	AdaBoost, Bagging Classifier (BAG), Bernoulli Naïve bayes (BNB), Decision Tree (DT), Extra Tree Classifier (ET), Gaussian Naïve bayes (GNB), GBoosting, K-Nearest Neighbor Classifier (KNN), Linear Discriminant Analysis (LDA), LR, MLP, Nearest Centroid (NC), Passive Aggressive Classifier (PA), Quadratic Discriminant Analysis (QDA), RF, Ridge Classifier, SVM and Ensemble Voting (EV).	LRRK2: 264 GBA: 129	LRRK2: 0.98 GBA: 0.90
El-Gawady et al. (2022)	AD	Gene expression		GEO	SVM, RF, LR, AdaBoost	1,157	0.975
Abbas et al. (2022)	AD	DNA methylation, gene expression		GEO	AE-SVM, RF, NB, XGBoost	697	0.9182
Bi et al. (2021)	PD	SNPs	fMRI	PPMI, ADNI	clustering evolutionary random neural network ensemble (CERNNE)	104	0.886

To achieve the best performance of gene expression-based AD diagnosis, several studies compared multiple ML models. For example, El-Gawady et al. (2022) applied Support Vector Machine (SVM), Random Forest (RF), Logistic Regression (LR), and AdaBoost models on gene expression. They used a meticulous gene selection (GS) algorithm to identify the most relevant genes and identified that the SVM as the most accurate model. In validation with 157 unseen cases, the SVM classifier achieved impressive metrics with 0.972 area under the receiver operating characteristic curve (AUC) and 0.975 accuracy. In PD, 5–10% of cases have a genetic origin with mutations identified in several genes such as leucine-rich repeat kinase 2 (LRRK2) and glucocerebrosidase (GBA). Hence, Hajianfar et al. (2023) proposed a hybrid ML system consisted of 11 feature extraction algorithms (FEA), 10 feature selection algorithms (FSA) and 22 classification algorithms (CA) for PD prediction based on three modalities: convolutional imaging data, clinical data, and radiomics features. For radiomics features (RF), they picked 264 and 129 patients with known LRRK2 and GBA mutations status from PPMI database. Consequently, they obtained 513 features, including 55 clinical features (CFs), 28 conventional imaging features (CIFs), and 215 RFs extracted from each ROI of DAT-SPECTimage (in total, 430 for both ROIs) using their Standardized Environment for Radiomic Analysis (SERA) software. Lin et al. (2022) utilized RF to identify blood-sample gene biomarkers for predicting stable mild cognitive impairment (sMCI) patients. Using two datasets from ADNI, the researchers identified 29 gene biomarkers (31 probes) that were effective in predicting sMCI. The RF classifier achieved AUC of 0.841. To address the high dimensionality and low sample size issue, Abbas et al. (2022) implemented dimensionality reduction that improved accuracy and AUC of multiple ML models. Furthermore, integrating DNA methylation and gene expression data significantly enhanced prediction performance, leading to a 9.5% improvement in accuracy and a 10.6% increase in AUC compared to state-of-the-art methods.

Alatrany et al. (2023) focuses on improving the classification of AD by identifying relevant SNP biomarkers using a novel approach that combines deep transfer learning (TL) and CNNs. The workflow involves training CNNs on multiple genome-wide association studies (GWAS) datasets, including data from an animal population and two distinct human populations. Initially, CNNs were trained on a GWAS dataset from the ADNI, and the trained models were further refined through TL applied to additional GWAS datasets. This process involved customizing the TL models to extract a robust set of features, which were then classified using an SVM. The experimental results, which included multiple configurations, achieved an accuracy of 89%, significantly outperforming existing methods. The innovation lies in the strategic use of deep TL across diverse datasets to enhance SNP-based AD classification, leading to more accurate and reliable diagnostic predictions. Shigemizu et al. (2019) investigated potential microRNA (miRNA) biomarkers for DLB and developed a risk prediction model using serum miRNA expression data from 478 Japanese individuals. Several ML methods were applied, including penalized regression, RF, SVM, and gradient boosting decision tree (GBDT). The GBDT model, which used 180 miRNAs and two clinical features, achieved the best performance with an accuracy of 0.829 on an independent test set. Additionally, gene set enrichment analysis revealed six functional genes associated with DLB pathology, with BCL2L1 and PIK3R2 being statistically significant in gene-based association tests. The proposed model offers a promising tool for DLB classification and highlights potential miRNA-related biomarkers and pathways involved in DLB (Shigemizu et al., 2019).

Singh et al. (2019) leverages imaging data from the PPMI and ADNI, involving 2,540 subjects across five classes: AD, PD, MCI, scans without evidence of dopaminergic deficit (SWEDD), and HCs. Using an ML framework combining principal component analysis (PCA) for feature extraction, Fisher discriminant ratio for feature selection, and least-squares SVM for classification, the study achieved an 87.89 ± 3.98% accuracy in multiclass classification, with precision of 82.54 ± 8.85%. The binary classification accuracy reached 100%, demonstrating the system's potential for clinical diagnostics in neurodegenerative diseases. Bi et al. (2021) presents a multimodal fusion framework for detecting PD and analyzing its pathogenic factors using SNPs and fMRI. A novel ensemble learning model analyzes fusion features derived from correlation analysis between genes and brain regions, achieving an accuracy of 88.57% in classifying PD patient.

### 3.2 Deep learning in neurodegeneration

#### 3.2.1 Deep neural network

DL, as a subset of ML, is well-suited for handling large datasets, allowing it to identify more complex patterns from raw data and improve predictive accuracy (Mobarak et al., 2023). DL has shown remarkable success in diverse domains such as computer vision, natural language processing, and genomics (Yue et al., 2023). In the context of neurodegenerative disease diagnosis, DL demonstrates substantial potential in detecting early disease markers, predicting disease progression, and identifying patient subgroups based on imaging and molecular features (Zhou and Troyanskaya, 2015). The simplest DL model is the DNN, which is composed of multiple layers of interconnected nodes (neurons) that process and transform data to learn complex patterns and make predictions (Kufel et al., 2023). Park C. et al. (2020) utilized large-scale gene expression and DNA methylation data to develop a DNN model for predicting AD and the model demonstrated improved performance over conventional ML algorithms. Ponce de Leon-Sanchez et al. (2023) used a DNN to predict MS using 35 genetic biomarkers and achieved an accuracy of 89.65%. Ning et al. (2018) proposed a DNN to predict AD risk using genetic variants (i.e., SNPs) and neuroimaging data (i.e., MRI-derived brain morphometric measures) on ADNI database. Table 3 lists the studies that involve DL methods in neurodegenerative diseases.

**Table 3:** Overview of DL methods applied in neurodegenerative disease.

**References**	**Disease**	**Genomics**	**Image**	**Dataset**	**Feature selection/extraction**	**DL Method**	**Acc**	**AUC**
Jemimah et al. (2023)	AD	Genotyping data, gene expression		ADNI	KEGG pathway constraints, SHAP scores	DNNs	0.69	0.70
Ponce de Leon-Sanchez et al. (2023)	MS	Gene expression		GEO	kNN, Gaussian NB, C-SVC, decision tree	DNNs	0.8965	0.8603
Zhou et al. (2019)	AD	SNPs	PET, MRI	ADNI	Latent representation	DNNs	0.644	N/A
Kalkan et al. (2022)	AD	mRNA expression		GEO	Fisher distance, LDA	CNNs	0.842	N/A
Li L. et al. (2021)	AD	SNPs		ADNI	Quality control	ResNet	0.9878	
Jo et al. (2022)	AD	SNPs		ADNI		CNNs	0.75	0.82
Chung and Lee (2023)	AD	Gene expression		GEO, ADNI	Deep metric learning	1D-CNNs	0.652	0.877
Abdelwahab et al. (2023)	AD	Gene expression		GEO	PCA, SVD	CNNs	0.9660	N/A
Kim et al. (2021)	AD	SNVs, exon splicing data, RNA-seq		GEO	Functional note, GWAS	ResNet	NO	N/A
Varathan et al. (2023)	AD	Gene expression		ROSMAP, HPRDPPI	GLRP	Graph-CNNs	0.79167	0.76588
Rohini et al. (2024)	AD	DNA sequence		ADNI	N/A	CNNs	N/A	N/A
Basheera and Sai Ram (2019)	AD		MRI	ADNI	ICA	CNNs	0.9047	0.885–1
Ren et al. (2019)	AD		MRI	ADNI	RF	SBPCNNs, SACNNs, MSCNNs	0.9375	0.93
Padole et al. (2020)	AD		Rs-fMRI	ADNI, Erdös-Rényi, airfoil, Minnesota	Graph coarsening	GCNNs	0.993	N/A
Huang et al. (2019)	AD		MRI, PET	ADNI	3D-CNNs	3D-CNNs	0.769–0.901	0.9269
Jo et al. (2020)	AD		PET	ADNI	CNNs, LRP	CNNs	0.908	N/A
Feng et al. (2020)	AD		MRI	ADNI	3D-CNN-SVM	3D-CNN-SVM	0.9574	0.998
Li Y. et al. (2021)	AD	SNPs	MRI	ADNI, AIBL	PCA	CNNs	0.992	0.78
Venkatasubramanian et al. (2023)	AD		MRI	ADNI	CNNs	CNNs	Segmentation: 0.971 Multi-class: 0.93 Binary: 0.96	N/A
Qiu et al. (2022)	AD		MRI	NACC, ADNI, AIBL, FHS, LBDSU, NIFD, OASIS, PPMI	CatBoost	CNNs	0.896	0.974
Mahmud et al. (2023)	AD		MRI	ADNI	CNNs	CNNs	0.882	0.945
Mohi ud din dar et al. (2023)	AD		MRI	ADNI	CNNs	CNNs	0.9622	N/A
Babu et al. (2022)	AD		MRI		GLCM, Haralick features, genometrics haralick features	SVM, CNNs	0.95192	N/A
Kim and Lee (2023)	AD		MRI	KACD	LFA	CNNs	0.986	N/A
Al-Adhaileh (2022)	AD		MRI	KACD	AlexNet	AlexNet	0.9453	0.991
El-Assy et al. (2024)	AD		MRI	ADNI	CNNs	CNNs	0.99	0.9994
Khagi and Kwon (2020)	AD		MRI, PET	ADNI	CNNs	CNNs	0.9459	N/A
George et al. (2022)	AD	DEGs	Gene corresponding rat brain image	GEO	Functional enrichment, pathway analysis	CNN-VGG16	0.61	N/A
Venugopalan et al. (2021)	AD	SNPs	MRI	ADNI	kNN, SVM, decision tree, RF	3D-CNNs	0.89	N/A
Chakraborty et al. (2023)	AD	SNPs	MRI	ADNI	CNNs	CNNs	0.88	0.72
Amini et al. (2022)	AD	SNPs	PET	ADNI	PCA	KNNs, LDA, SVM, CNNs	0.911	
Bi et al. (2022)	AD	SNPs	fMRI	ADNI	Full-gradient saliency graph mechanism, weight combination between adjacent layers	Graph CNNs	0.8936	0.85
Mahendran and Durai Raj Vincent (2022)	AD	DNA methylation		GEO	LASSO, SVM, AdaBoost	RNNs	0.887	0.876
Park J. et al. (2020)	AD	RNA-seq		GEO	Latent space interpolation, pathway analysis	GANs	N/A	N/A
Shen et al. (2019)	AD		PET	ADNI	PCA	DBNs	0.866	N/A
Chakraborty et al. (2023)	PD		MRI	PPMI	Atlas-based segmentation	MLP	0.953	N/A
Reyes et al. (2022)	PD	SNPs		PPMI, PDBP	PCA, linear regression	Transformer	N/A	0.708 - 0.581
Chen et al. (2024)	PD		MRI	Shanghai Jiao Tong University	Transformer	Transformer	0.83	0.90
Salvi et al. (2023)	Prion disease		WSIs	DOMP	Texture analysis (TA)	Vision transformer	0.937 ± 0.068	N/A
Mahendran et al. (2021)	AD	Gene expression		GEO	Wrapper based on PSO, AE	DBN	0.9678	N/A
Chen et al. (2022)	AD	DNA methylation		ADNI	AE	LSTM autoencoder	N/A	0.996
Alamro et al. (2023)	AD	Gene expression		GEO	LASSO, ridge regression	DNN, CNNs	N/A	0.979
Maj et al. (2019)	AD	Gene expression		ADNI	AE	SVM, FNN, CNNs, RNNs	N/A	0.953
McKeever et al. (2023)	ALS	RNA sequences, RBP expression profiles		SRA	RF	CNNs, attention	N/A	0.97
Kamal et al. (2021)	AD	Gene expression	MRI	Kaggle, OASIS-3, GEO	CNN, spinal net	CNN, Xboost, KNN, SVC, LIME	0.976	N/A
Wang et al. (2022)	AD	SNPs	MRI	ADNI	3D-CNN, transformer encoder	MLP	0.8378	0.924
Ying et al. (2021)	AD	SNPs	MRI	ADNI	CNN, MLP	CNN, MLP	0.961	0.935
Qiang et al. (2023)	AD	SNPs	sMRI	ADNI	Patch-CNN, MLP, self-attention	Patch-CNN, MLP, self-attention	0.93	N/A
Lyu et al. (2021)	AD	RNA expression	MRI, rs-fMRI	ADNI	LSTM, GCN-attention, FCN	LSTM, GCN-attention, FCN	0.823	N/A
Parvin et al. (2024)	AD		MRI	OASIS, Kaggle, GEO	Knowledge graph	SVM, CNN, XGBoost	N/A	0.98
Song et al. (2023)	PD		MRI	SMC	CNN based V-Net	Vision transformer	N/A	0.91–0.94

RBP, RNA-binding protein; KACD, Kaggle Alzheimer's classification dataset; SMC, Samsung Medical Center; SRA, Sequence Read Archive; DOMP, Diagnosis and Monitoring of Prion Diseases (DOMP); GLCM, Gray Level Co-Occurrence Matrix; SVC, Support Vector Classification; NB, Naïve Bayes; GLRP, Guided Layer-wise Relevance Propagation; LDA, Linear Discriminant Analysis; DEG, Differentially Expressed Genes; PDBP, Parkinson's Disease Biomarkers Program; PSO, Particle Swarm Optimization; LIME, Local Interpretable Model-Agnostic Explanation.

#### 3.2.2 Convolutional neural networks

CNN is a type of DL model designed to automatically and efficiently extract spatial features from input data, typically images, using convolutional layers to detect patterns like edges, textures, and shapes for tasks such as image recognition and classification (Yamashita et al., 2018). It has emerged as a powerful DL tool in neurodegenerative disease research due to their ability to effectively process and analyze high-dimensional neuroimaging data.

Jo et al. (2022) achieved an AUC of 0.82 by combining CNN with a Sliding Window Association Test (SWAT) to identify AD-related SNPs. The model successfully identifying the APOE region, known to have high correlations with AD, as a significant genetic locus for AD. Another study employed a CNN in protein subcellular localization, where CNN acted as a feature extractor integrated with XGBoost, to identify protein subcellular localization based on gene sequence data (Pang et al., 2019). Similarly, CNNs were used in combination with k-means clustering for analyzing microarray gene expression data in AD prediction and achieved 92.9% accuracy (AL-Bermany and AL-Rashid, 2021). Abdelwahab et al. (2023) also utilized microarray data and a seven-layer CNN for AD prediction, achieving accuracy rates of 96.60 and 97.08% with Principal Component Analysis (PCA)-CNN and Singular Value Decomposition (SVD)-CNN models, respectively, indicating strong performance by CNNs when combined with gene selection techniques. Further applications of CNNs extend to DNA methylation data analysis for early AD diagnosis. Babichev et al. (2025) demonstrated that CNN could be effectively combined with gene ontology analysis, clustering, and Bayesian optimization for diagnosing both AD and cancer, achieving classification accuracy rates of 89.8 and 91.8% for AD subsets.

CNNs also show promise in predicting disease conversion from MCI to AD. A study proposed a deep CNN model for processing gene expression data, specifically focusing on identifying transcription factors linked to disease progression (Rohini et al., 2024). In exploring the cellular mechanisms underlying AD, Wu et al. (2024) integrated single-cell RNA sequencing (scRNA-seq), Weighted Gene Co-expression Network Analysis (WGCNA), and CNN to identify critical genes and microglial subclusters involved in AD pathology. This approach pinpointed nine hub genes, including USP3, which were identified as potential therapeutic targets. Kim et al. (2021) applied a CNN model called SpliceAI, combined with GWAS, to identify SNVs and abnormal splicing in the phospholipase C gamma-1 (PLCγ1) gene associated with AD progression. McKeever et al. (2023) developed alternative polyadenylation (APA)-Net for ALS. The model demonstrated strong correlations in predicting APA log-fold change values across different ALS subtypes, providing insights into cell-type-specific APA profiles.

Additionally, CNN regression models were used to explore the impact of AD-related genetic variants on cis-regulatory elements across different cell types. These models revealed the significant role of peripheral immune cells in AD predisposition (Ramamurthy et al., 2022). Kalkan et al. (2022) transformed one-dimensional gene expression data into two-dimensional images, which were then classified using CNN, achieving high accuracy and AUC scores in AD prediction. Similarly, George et al. (2022) combined gene expression analysis with histopathological brain image analysis using CNN-VGG16, identifying key differentially expressed genes in AD rat models and demonstrating strong predictive power. Basheera and Sai Ram (2019) used CNNs to classify AD by extracting gray matter from 1,820 T2-weighted brain MRI volumes, achieving 90.47% accuracy. Similarly, Ren et al. (2019) developed CNN variants focused on specific brain regions, leveraging RF for feature selection and maintaining model interpretability. Feng et al. (2020) extended this with 3D-CNNs on ADNI MRI data for ternary classification, advancing volumetric image analysis. Li L. et al. (2021) used genotype data from 1,461 ADNI participants to develop a DL genomics (DLG) model based on the ResNet framework for classifying AD, MCI, and HC. Validated with 5-fold cross-validation, the DLG model achieved 98.78% accuracy in distinguishing AD from HC, outperforming traditional GWAS (71.38%). The study also identified novel genetic biomarkers for AD progression.

Venkatasubramanian et al. (2023) introduces a 3D-ResNet that uses structural MRI data to simultaneously perform hippocampus segmentation and AD classification. The model, optimized by the deer hunting optimization (DHO) technique, was tested on ADNI MRI datasets. It achieved 97.1% accuracy in segmentation with a 96% accuracy for binary classification and 93% for multi-class classification, demonstrating its effectiveness in early AD detection. Qiu et al. (2022) integrated multimodal data (MRI and clinical information) using a hybrid DL framework, achieving performance comparable to clinicians in diagnosing AD and non-AD dementias (nADDs). Babu et al. (2022) combined advanced feature extraction techniques with a hybrid CNN-SVM model for early AD detection, especially at the MCI stage. Kim and Lee (2023) introduced an ensemble CNN combining brain shape analysis with VGGNet-based image classification, achieving 98.6% accuracy on MRI datasets. This model integrated both structural and image features, enhancing classification performance. Given the prevalence of unbalanced data in research, both oversampling and upsampling techniques are common preprocessing methods for image data prior to input in CNNs for AD stage classification.

Several studies addressed class imbalance. Mahmud et al. (2023) utilized data augmentation and oversampling techniques to enhance CNN performance for binary classification, while Mohi ud din dar et al. (2023) applied upsampling for improved AD diagnosis. Al-Adhaileh (2022) compared AlexNet and ResNet50 using transfer learning on Kaggle MRI data, with AlexNet outperforming ResNet50, achieving 94.53% accuracy. Finally, a multi-filter CNN architecture from the ADNI dataset (El-Assy et al., 2024) achieved over 99% accuracy in multi-category classification of AD, highlighting its potential for early detection and personalized treatment. Jo et al. (2020) employed a DL framework integrating a 3D CNN with layer-wise relevance propagation (LRP) to analyze tau PET scans, achieving 90.8% accuracy in classifying AD from CN individuals. LRP pinpointed key regions, including the hippocampus and parahippocampus, and AD probability scores correlated with tau deposition in the medial temporal lobe in MCI participants. This approach demonstrates potential for early AD detection using tau PET imaging. Huang et al. (2019) proposed a CNN model that integrates multimodal information from T1-weighted MRI and FDG-PET images of the hippocampal area to diagnose AD. The CNN achieved accuracies of 90.10% for CN vs. AD, 87.46% for CN vs. pMCI, and 76.90% for sMCI vs. pMCI in ADNI data, demonstrating its effectiveness and the advantage of combining multiple imaging modalities. Khagi and Kwon (2020) introduced divNet, a simple encoder-based CNN designed for AD diagnosis using MRI and PET imaging data. The research examined the transition from 2D to 3D CNN architectures, focusing on how variations in filter size and stride affect feature extraction. A novel two-stage graph coarsening method for graph CNN (GCNNs) was proposed in another study by Padole et al. (2020) This method combines graph wavelet transform (GWT)-based features with an optimization problem to enhance coarsening by maximizing topological similarity. Applied as a pooling operation within a modified GCNN architecture, this approach was used for early AD detection through graph signal classification. It demonstrated superior performance in both general graphs coarsening and as a pooling operator in GCNNs.

**Figure 2:**
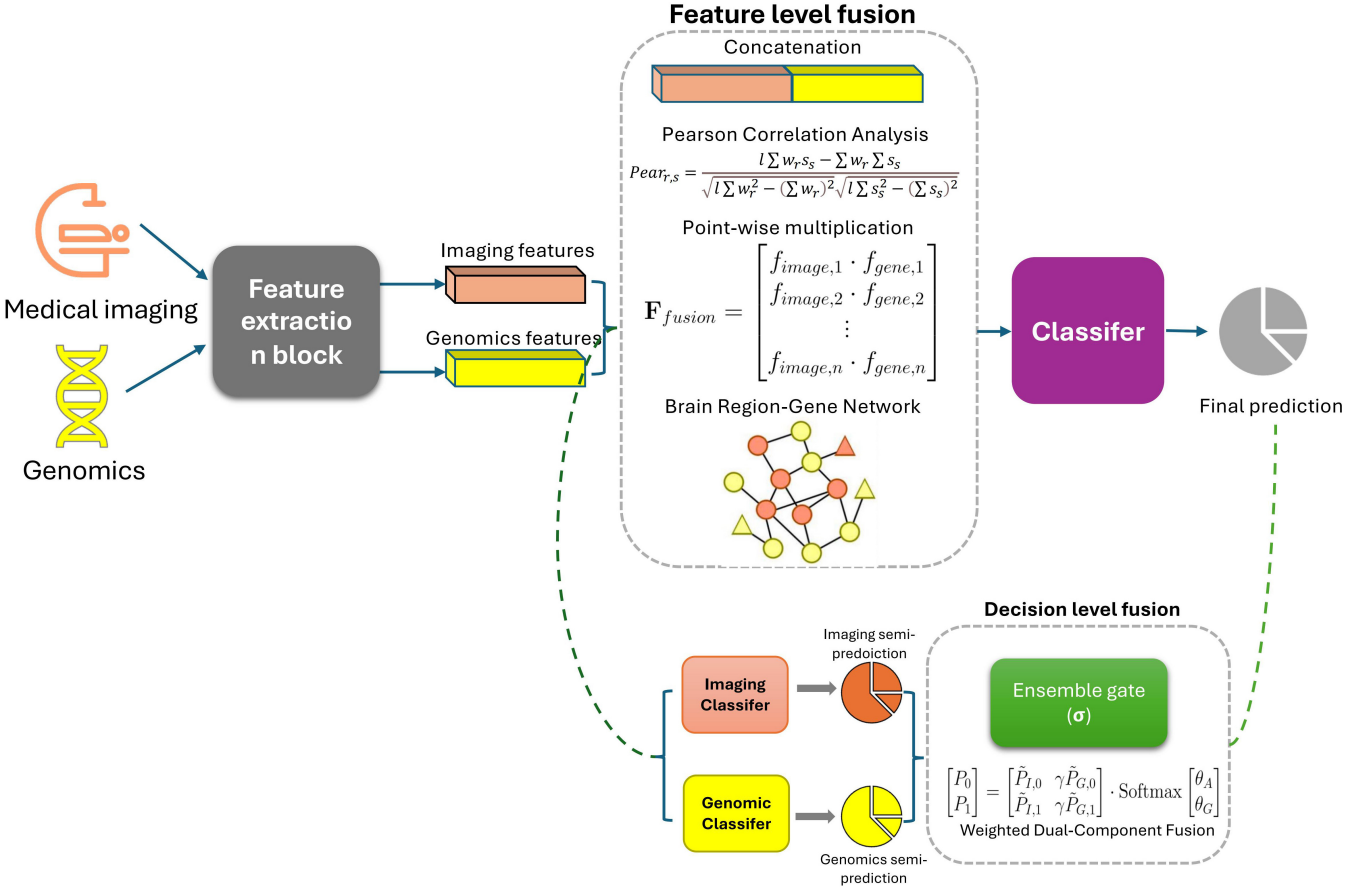
Current multi-modal fusing methods in deep learning-based radiogenomics workflow.

As CNNs excel in medical imaging for neurodegenerative disease diagnosis, their integration with genomics data in radiogenomics offers deeper insights into disease mechanisms, enhancing precision diagnostics. Figure 2 shows the common ways of integrating radiomics with genomics.

Amini et al. (2022) proposed a study using SNPs and PET imaging data to explore their causal relationship and classify AD. Models applied were k-nearest neighbor (KNN), SVM, linear discrimination analysis (LDA), and CNN. CNN achieved the highest classification accuracy at 91.1%, indicating that KNN and CNN are effective for diagnosing AD, while LDA and SVM showed lower accuracy. The suggested SNPs were more strongly associated with quantitative traits than ApoE gene SNPs. Bi et al. (2022) introduced a novel DL approach, the Feature Aggregation Graph Convolutional Network (FAGCN), to analyze relationships between brain region activities and gene expression patterns for detecting AD. The model integrates fMRI data and genetic data (SNPs) to construct brain region-gene networks. It then applies a series of graph convolutions (1st-order and 2nd-order) to capture complex interactions within the network. The results are processed through fully connected layers and a softmax classifier to determine the classification of samples. This method also uses a full-gradient saliency graph to identify and highlight pathogenetic brain regions and risk genes related to AD. The model shows superior performance compared to traditional methods in identifying AD-related features, providing a more detailed understanding of the disease's development. Venugopalan et al. (2021) presents a CNN framework that integrates imaging (MRI), genetic (SNPs), and clinical test data to classify patients into AD, MCI, and CN groups for the ADNI dataset, the CNN outperformed traditional ML like SVMs and decision trees. Zhou et al. (2019) leveraged multimodal neuroimaging data (MRI, PET) and genetic data (SNPs) from the ADNI database to diagnose AD and MCI. The researchers developed a three-stage DL model that effectively managed the heterogeneity of different data types. The model first learned independent latent features for each modality, then combined these into joint features, and finally fused them to predict diagnostic outcomes. By using multiple scanning time points, the model outperformed existing methods in accuracy and AUC, though specific values were not disclosed. Chakraborty et al. (2023) utilized CNN models on three-dimensional MRI data to automatically extract brain features for GWAS studies aimed at identifying genetic variants linked to brain atrophy in AD. By applying this method to ADNI data, the researchers identified several SNPs associated with neurodegenerative and mental disorders, including AD, depression, and schizophrenia.

#### 3.2.3 Hybrid DL models

Several other types of DL models are often applied to neurodegenerative diseases in a hybrid approach, combined with CNN or ML modules. This section will introduce these DL models and explain how they are integrated with CNN or ML modules in neurodegenerative disease studies.

Hochreiter and Schmidhuber (1997) introduced the Recurrent Neural Networks (RNN) architecture, a type of DL designed to handle sequential data by maintaining a hidden state that captures information from previous inputs. RNNs have become a significant tool in DL, particularly for sequence data analysis. An RNN comprises a network that iteratively processes input data and updates its hidden state, allowing it to capture temporal dependencies. However, traditional RNNs suffer from issues like vanishing gradients, making it challenging to learn long-range dependencies. To address these issues, Hochreiter and Schmidhuber developed the Long Short-Term Memory (LSTM) network, which includes a cell state and gated mechanisms (input, forget, and output gates) to regulate the flow of information. The LSTM's ability to maintain a stable cell state allows it to capture long-term dependencies effectively. Similarly, Abbas et al. (2022) introduced the Gated Recurrent Unit (GRU), a variant of the LSTM that simplifies the architecture by combining the input and forget gates into a single update gate and using a reset gate to manage information flow. Both LSTM and GRU have been widely adopted due to their improved capability in learning long-term dependencies compared to traditional RNNs.

Goodfellow et al. (2014) developed the Generative Adversarial Network (GAN) model, a type of generative model that uses an adversarial approach. GANs have since become a prominent area in DL, particularly for computer vision and image processing tasks like image generation (Lan et al., 2020; Alqahtani et al., 2021). A GAN comprises two networks: a generative network that creates fake data based on a latent variable, and a discriminative network that discerns real data from fake data (Arjovsky et al., 2017). These networks compete against each other in an adversarial manner. To address training instability, Arjovsky et al. (2017) introduced the Wasserstein GAN (WGAN), which uses the Earth Mover's distance (Wasserstein distance) to measure distribution differences, as opposed to the original GAN's Jensen–Shannon divergence, which is problematic when distributions do not overlap (Arjovsky et al., 2017). Additionally, the conditional Wasserstein GAN (cWGAN) variant includes a gradient penalty term to enhance performance (Arjovsky et al., 2017; Gulrajani et al., 2017). Deep Belief Networks (DBN) represents a category of DL frameworks composed of multiple layers of hidden or latent variables. Each layer in a DBN captures the correlation information from the activities of hidden or latent variables in the preceding layer (Akhavan Aghdam et al., 2018; Hinton et al., 2006). Typically, each layer's foundational component is a restricted Boltzmann machine (RBM), which is a type of two-way undirected graphical model (Akhavan Aghdam et al., 2018; Hinton et al., 2006). A variant known as the sparse-response DBN has been introduced, utilizing rate-distortion theory. This approach encodes the original data into a sparse dataset using fewer bits, thereby enhancing performance (Ji et al., 2014). Autoencoder (AE) comprises an encoder and a decoder unit, typically implemented using fully connected DNN layers. It has several notable variants such as Variational AE (VAE; Shui et al., 2021), Adversarial AE (AAE) model (Mazurowski, 2015), and Adversarial Variational Autoencoder (AVAE; Jack et al., 2008). A graph neural network (GNN; Zhou et al., 2020) is a type of DL model designed to work directly with graph-structured data by learning the relationships and dependencies between nodes and edges, making it ideal for tasks like node classification, link prediction, and graph classification.

Maj et al. (2019) analyzed genotype data from AD, MCI patients, and controls using tissue-specific cis-eQTL models. A VAE was used for feature extraction and SVM for classification, with RNNs as the best-performing model. Although ACC and AUC were not reported, the study highlighted inflammatory processes in gut-brain axis tissues and emphasized the benefit of integrating unsupervised and supervised learning for high-dimensional omics data. Akkaya and Kalkan (2023) introduced One2MFusion which integrates gene expression data with their 2D representations for AD classification. The approach used CNN to process 2D gene images and DNN to analyze gene sequences with an increased AUC of 0.91 for AD vs. NC and 0.88 for MCI vs. NC. Kamal et al. (2021) proposed a study classified AD using both MRI images and microarray gene expression data. In their study, SpinalNet (Kabir et al., 2023) and CNN were applied to MRI images, while KNN, SVM, and XGBoost were used on gene expression data. SpinalNet, inspired by the human nervous system, efficiently processes inputs hierarchically, reducing computation and overfitting. CNN achieved 97.6% accuracy, outperforming SpinalNet by 10.96%, and SVM showed the highest accuracy 82.4% for gene expression data. Mahendran and Durai Raj Vincent (2022) developed an emhanced RNN for the early diagnosis of AD using DNA methylation data from the GEO database. To manage the high-dimensional data, three embedded feature selection methods—LASSO, SVM with regularization, and AdaBoost—were compared. The proposed approach significantly outperformed existing models, including CNN, RNN, and DRNN, in classification accuracy.

Chen et al. (2022) developed two multi-task deep AEs—one based on a convolutional AE and another based on a LSTM AE—to predict AD progression using DNA methylation data from peripheral blood. The deep AEs were designed to learn compressed feature representations by jointly minimizing reconstruction error and maximizing prediction accuracy. Benchmarking on longitudinal DNA methylation data from ADNI, the proposed models outperformed state-of-the-art ML approaches in predicting AD progression and reconstructing temporal DNA methylation profiles. Ying et al. (2021) developed a multimodal model that combines brain MRI and SNP data for AD diagnosis. The model integrates a 2D CNN for MRI analysis and a multi-layer perceptron (MLP) for processing SNPs. Tested on the ADNI dataset, the model achieved 93.5% AUC. Qiang et al. (2023) combined CNN and RNN to enhance AD diagnosis based one sMRI, clinical data, and APOE genetic data. It achieved 93% accuracy for AD vs. MCI and 82.4% accuracy for MCI vs. NC on the ADNI database. Mahendran et al. (2021) combined AE and DBN for AD diagnosis using the GSE5281 dataset from GEO. Lyu et al. (2021) developed a cross-datatype deep fusion model (CDF-Model) to classify MCI patients from NC. The model integrates a RNN for multimodality brain imaging data and a DNN for processing gene expression data and achieved an overall accuracy of 82.3% on ADNI dataset. Afshar et al. (2023) revealed that risk genes underlying AD were more connected in microglia through creating a multimodal DL model combining GNN and CNN. Similarly, Parvin et al. (2024) also combined GNN with CNN for multimodal data integration for AD classification. Explainable AI (XAI) techniques such as layer-wise relevance propagation and submodular pick local interpretable model-agnostic explanations (SP-LIME) are used for model interpretation in this study. Wang et al. (2022) used 3D CNN to process 3D MRI inputs and RNN to handle genetic sequence data. The combined embeddings from these two modalities are further processed through a MLP classifier. IGnet achieved a classification accuracy of 83.78% and an AUC of 0.924 on ADNI data.

#### 3.2.4 Transformers

Transformer, introduced by Vaswani et al. (n.d.) in 2017, is a groundbreaking DL architecture. Unlike traditional models like RNNs, which process data sequentially, the Transformer employs a self-attention mechanism that allows it to analyze entire input sequences in parallel. This enables the model to capture relationships between words regardless of their positions in the text. To maintain the order of words, the Transformer incorporates positional encoding. Additionally, it uses multi-head attention to focus on different parts of a sentence simultaneously, enhancing its ability to understand complex language structures. The model's architecture typically includes an encoder and decoder, making it highly effective for tasks like machine translation.

Reyes et al. (2022) applied Transformer on genotype data (SNP) for PD diagnosis. The model is designed to effectively capture complex global feature interactions that are challenging for traditional methods like polygenic risk scores and standard ML approaches. By leveraging the attention mechanism in Transformer, they noticed improved classification accuracy and enhanced interpretability through the visualization of learned SNP-SNP associations. Chen et al. (2024) introduces a novel DL model called the Segmentation-Transformer-Age-Network (STAN) to predict brain age using Quantitative Susceptibility Mapping (QSM) data. The STAN model utilizes a two-stage network architecture: the first stage focuses on extracting informative features from QSM data through segmentation training, while the second stage integrates these features to predict brain age. The study was conducted using QSM images from 712 healthy participants (548 for training and 164 for testing). The model achieved a high accuracy in predicting brain age, with a mean absolute error (MAE) of 4.124 years and a coefficient of determination (R^2^) of 0.933. Song et al. (2023) applied Vision Transformer (ViT) to segment brain MRI scans for the diagnosis of PD with a accuracy large than 0.85 and were 300 times faster than DNN. Watanabe et al. (2021) utilized a transformer-based generative model to generate SPECT images characteristic of PD. The model was able to generate inferior slices of a 3D volume from a few superior slices and transform HC SPECT images into PD-like images.

## 4 Challenges and future direction

The quality of datasets remains a significant challenge in neurodegenerative disease radiogenomics research. While numerous publicly available datasets exist, as highlighted in Table 1, they do not fully represent the central dogma of molecular biology. Comprehensive, high-quality data are required at all stages of gene expression, from DNA transcription to RNA translation and protein synthesis, to better understand the molecular mechanisms driving these diseases. Additionally, there is a notable imbalance in available data, particularly a lack of cases representing early-onset AD and rarer dementias, such as FTD and VD. This imbalance hinders the ability of models to generalize and accurately differentiate between subtypes, such as amnestic vs. non-amnestic MCI. Furthermore, the issue of unpaired data complicates multimodal integration; for example, PET data is far less prevalent than MRI or SNP data, limiting comprehensive radiogenomic analyses. Addressing these data quality and availability gaps is essential for improving the accuracy of models and advancing neurodegenerative disease research. More complete, well-annotated datasets are crucial for the future of radiogenomics in this field.

Multi-center and international collaborations, such as the ADNI, play a crucial role in addressing data imbalance and heterogeneity by aggregating larger and more diverse demographic datasets. Establishing standardized protocols for data collection—including imaging, genomic sequencing, and clinical annotations—can further enhance dataset consistency and comparability across studies. Generative models, such as GANs and diffusion models (Ho et al., 2020), offer significant promise for addressing data imbalance, particularly in high-dimensional datasets like imaging or genomics. By generating realistic synthetic data that mirrors the distribution of underrepresented classes, these models help mitigate class imbalance and improve model training. For example, synthetic samples generated for rare classes can balance datasets, ultimately enhancing model performance and robustness.

To tackle heterogeneity in multimodal data, the primary challenge is extracting relevant features from different modalities (e.g., MRI, PET, genomics) while preserving critical information. Techniques such as cross-modal attention mechanisms (Zhang et al., 2023) and multi-view learning (Liu et al., 2017) enable the integration of features across modalities, allowing models to focus on the most salient aspects and learn meaningful representations with minimal information loss. Shared latent representation approaches, such as canonical correlation analysis (CCA) and multimodal variational autoencoders (VAEs), align features from multiple data sources in a common latent space while retaining the unique characteristics of each modality.

Data normalization techniques, such as min-max scaling or z-score normalization, are essential for preprocessing data with varying ranges and distributions, ensuring compatibility across sources. For datasets with insufficient size to train models from scratch, transfer learning is an effective strategy. Models pretrained on large datasets (e.g., ImageNet for images) can be fine-tuned on smaller datasets, enabling generalization to new domains. Additionally, federated learning (Rastogi et al., 2024) addresses privacy concerns and institutional data disparities by enabling decentralized model training across distributed datasets without sharing raw data, preserving privacy while leveraging diverse sources. To further address data heterogeneity, domain adaptation techniques offer a promising solution by bridging the gap between source and target domains with differing distributions, such as those arising from multi-modal or cross-institutional variations. For instance, unsupervised domain adaptation (Yu et al., 2024) minimizes domain discrepancies through methods like adversarial learning, aligning feature representations between datasets. In multi-modal scenarios, these techniques enable robust feature extraction and integration while preserving each modality's unique characteristics, ensuring models generalize effectively across diverse datasets.

Current research indicates that most studies predominantly employ feature-level fusion for multimodal data integration in Figure 2. This approach is favored because it can capture richer inter-modal relationships, provide high-resolution data integration, and enhance model generalization. However, the increased feature dimensionality demands significantly higher computational resources and exacerbates issues related to data imbalance across modalities. As a result, there is ample opportunity to explore decision-level fusion methods, which offer better adaptability to data heterogeneity and lower computational complexity. Investigating such approaches could lead to more efficient, scalable models that maintain robust performance despite modality-specific variations or incomplete data. Balancing the strengths of both fusion strategies will be crucial to advancing the field and optimizing multimodal learning systems for practical applications.

Another significant challenge is the gap between AI-enhanced models and their translation to real-world clinical applications. Biomarkers identified in AI-based radiogenomics studies still require rigorous experimental validation before they can be confidently used in clinical practice. Without this, their clinical utility remains speculative. Additionally, the limited interpretability of DL models is a persistent issue. While XAI techniques have been developed to improve model transparency, the adoption of XAI tools in radiogenomics for neurodegenerative diseases is still limited, highlighting an opportunity for improvement. To address this gap, future studies should prioritize the integration of XAI tools into their workflows, ensuring that model decisions are both interpretable and clinically actionable. For example, model-agnostic methods such as SHapley Additive exPlanations (SHAP) and Local Interpretable Model-agnostic Explanations (LIME) can quantify feature importance for genomic and imaging inputs, helping to identify the most critical variables influencing predictions (Liu et al., 2022). Similarly, Gradient-weighted Class Activation Mapping (Grad-CAM) is particularly useful in radiogenomic imaging, allowing researchers to visualize key image regions contributing to the model's decisions. These techniques enable researchers to interpret both high-dimensional genomic data and imaging features in a more meaningful way, bridging the gap between model predictions and clinical understanding. Moreover, methods like Layer-wise Relevance Propagation (LRP) can be employed to provide detailed explanations of how individual layers in a neural network contribute to the final decision, offering greater transparency for multi-modal applications. These tools should be incorporated not only during the evaluation phase but also during model development, enabling iterative refinements to improve interpretability and performance simultaneously. Some studies continue to rely on handcrafted features from regions of interest (ROI), while interpretable, may fail to capture the full complexity of structural and functional brain data. Combining these traditional approaches with DL-based methods that leverage XAI tools could strike a balance between interpretability and the ability to detect subtle, high-dimensional patterns, enhancing generalizability across diverse patient populations. Bridging the gap between AI models and their clinical applicability will require not only enhanced interpretability and validation but also standardized practices in data collection and processing. AI models trained on diverse, high-quality datasets that reflect the real-world heterogeneity of neurodegenerative diseases will be better equipped to make clinically relevant predictions. Incorporating XAI tools throughout the development pipeline can improve trust and understanding, making it easier for clinicians to adopt AI-driven tools in everyday practice.

Since radiogenomics studies involve integration multi-modal image data and multi-level genomics data, an effective integration strategy is needed. Simple concatenation of features—often applied at the hidden layers or the final stages of DL models—is commonly used but may not fully exploit the complex interactions between imaging and genomic data. More advanced integration techniques, such as Bayesian tensor factorization (Liu et al., 2022) and cross-modal self-attention (Zhang et al., 2023), offer promising avenues for enhancing the precision of these models. Bayesian tensor factorization can extract latent factors from three dimensions tensors constructed from the RNA-sequencing expression, copy number variation, and DNA methylation data through latent factor decomposition. It also automatically selects the optimal tensor rank, a critical step for capturing and understanding the multi-omics features of patients. Cross-modal self-attention mechanisms integrate imaging features, such as MRI and PET data, with cerebrospinal fluid (CSF) features using a transformer encoder based on self-attention. In this process, imaging features act as the key and value vectors, while CSF features serve as the query input, enabling the model to capture potential relationships across modalities. In addition to the power in multi-modal fusion, the cross-modal self-attention mechanisms also play an important role in addressing the overlapping imaging features between the different neurodegenerative disease. This dynamic focus helps disentangle overlapping features by highlighting the distinct relationships between modalities. Similarly, contrastive learning aligns representations from different modalities in a shared latent space, encouraging the model to emphasize the differences and reduce redundancies between them, further improving feature separation and interpretability. Despite the potential of these advanced methods, there is still a lack of standardized, robust tools for multimodal integration in radiogenomics. The development of new tools that incorporate state-of-the-art integration techniques is critical to advance the field. Additionally, hybrid models that leverage both feature-level and decision-level fusion might offer a more nuanced understanding of the complex relationships between genomic alterations and imaging phenotypes, especially in the context of neurodegenerative diseases. The introduction of such tools could significantly improve the model's ability to generalize across different datasets and patient populations, leading to more accurate predictions and better clinical utility. To address this gap, future research should focus on developing scalable, interpretable, and clinically relevant integration frameworks that handle the inherent heterogeneity and complexity of multimodal data. Hence, appropriate performance matrics should be considered after the model design. In addition to common matrics like AUC and accuracy, additional matrices such as F1-score, which balance precision and recall, can better show the robustness of models. Moreover, the Matthews correlation coefficient (MCC) is particularly useful for assessing generalizability of model, especially on imbalanced datasets. Researchers are also encouraged to use external validation to ensure that the model generalizes well to unseen data.

To mitigate overfitting in high-dimensional genomic data, several strategies can be employed. Data augmentation techniques, such as SMOTE and bootstrapping, help increase training sample diversity, reducing overfitting. Regularization methods like L1/L2 regularization and dropout prevent the model from fitting irrelevant features by penalizing complexity. Additionally, dimension reduction tools like PCA and UMAP reduce the feature space, retaining essential patterns while lowering the risk of overfitting. By integrating these approaches, researchers can improve model robustness and generalizability, ensuring better performance on diverse genomic datasets.

More advanced DL models, such as Mamba (Gu and Dao, 2024), a newly developed architecture, offer promising opportunities for neurodegenerative disease radiogenomics studies. Mamba is highly efficient, scaling linearly with sequence length and offering significantly faster inference compared to traditional models. This makes it particularly useful for tasks where long-term dependencies in data need to be modeled, such as in genomics or radiogenomics. For example, in neurodegenerative disease radiogenomics, Mamba can effectively integrate multimodal data (like MRI, PET, and genomic sequences) to identify patterns or biomarkers without the computational overhead often associated with other DL models. In addition to its computational efficiency, Mamba's lightweight design and low memory usage make it well-suited for deployment in resource-constrained environments, such as small medical institutions or remote areas with limited infrastructure. By enabling models to run effectively on less powerful hardware, such as mobile devices or edge computing systems, Mamba facilitates broader accessibility and real-time applications. Moreover, its parallel computation capabilities significantly reduce training and testing times, making it easier to develop pre-trained models tailored to specific tasks. For instance, Mamba excels in gray matter structure segmentation on T1-weighted brain MRI (Wei et al., 2024), while its variant, U-Mamba (Ma et al., 2024), addresses overlapping imaging features, further enhancing its versatility. By incorporating Mamba into neurodegenerative disease studies, researchers can take advantage of its efficient memory usage and selective information processing, enabling more accurate and scalable models.

## 5 Conclusion

In conclusion, AI-based radiogenomics holds significant promise for advancing the diagnosis, prognosis, and treatment of neurodegenerative diseases. While current research has successfully integrated genomic and imaging data to reveal important insights, much work remains to fully unlock the potential of these technologies. Challenges such as data quality, heterogeneity, and model interpretability must be addressed to ensure clinical applicability. Future efforts should focus on improving multimodal data integration strategies, enhancing model transparency through explainable AI, and validating identified biomarkers through rigorous clinical trials. As radiogenomics continues to evolve, it has the potential to revolutionize personalized medicine, offering earlier and more accurate diagnoses, as well as tailored therapeutic approaches, ultimately improving the quality of life for patients worldwide. This work reviewed current status of AI-based radiogenomics in neurodegenerative diseases, summarizing key model designs, performance metrics, publicly available data resources, significant findings, and future research directions. It provides a starting point and guidance for those seeking to explore this emerging area of study.
